# A role for cortical dopamine in the paradoxical calming effects of psychostimulants

**DOI:** 10.1038/s41598-022-07029-2

**Published:** 2022-02-24

**Authors:** Sharonda S. Harris, Sara M. Green, Mayank Kumar, Nikhil M. Urs

**Affiliations:** Department of Pharmacology and Therapeutics, University of Florida, 1200 Newell Dr, ARB-R5-140, Gainesville, FL 32610 USA

**Keywords:** Neuroscience, Psychology

## Abstract

Psychostimulants have a paradoxical calming effect in the treatment of attention deficit hyperactivity disorder (ADHD), but their mechanism of action is unclear. Studies using dopamine (DA) transporter (DAT) knockout (KO) mice have suggested that the paradoxical calming effect of psychostimulants might occur through actions on serotonin (5-HT) neurotransmission. However, newer non-stimulant drugs, such as atomoxetine and guanfacine, suggest that targeting the norepinephrine (NE) system in the prefrontal cortex (PFC) might explain this paradoxical calming effect. Thus, we sought to clarify the mechanism of this paradoxical action of psychostimulants. Our ex vivo efflux experiments reveal that the NE transporter (NET) blocker desipramine elevates both norepinephrine (NE) and dopamine (DA), but not 5-HT levels, in PFC tissue slices from wild-type (WT) and DAT-KO, but not NET-KO mice. However, the 5-HT transporter (SERT) inhibitor fluoxetine elevates only 5-HT in all three genotypes. Systemic administration of desipramine or fluoxetine inhibits hyperactivity in DAT-KO mice, whereas local PFC infusion of desipramine alone produced this same effect. In contrast, pharmacological NE depletion and DA elevation using nepicastat also inhibits hyperactivity in DAT-KO mice. Together, these data suggest elevation of PFC DA and not NE or 5-HT, as a convergent mechanism for the paradoxical effects of psychostimulants observed in ADHD therapy.

## Introduction

Attention deficit hyperactivity disorder (ADHD) affects at least 5–10% of children, with increasing diagnoses every year. The primary line of treatment for ADHD are the psychostimulant class of drugs, including amphetamine (Aderall) and methylphenidate (Ritalin). Several theories have been postulated on the paradoxical calming effect of psychostimulants in ADHD patients. However, after decades of research, their exact molecular and anatomical mechanism of action is not clear. Studies on the role of different monoamine transporters in the actions of psychostimulants have been inconclusive. Using the hyperactive dopamine (DA) transporter (DAT) knockout (DAT-KO) mice, studies have shown that administration of the 5-HT transporter (SERT) inhibitor fluoxetine or the 5-HT precursor 5-HTP, reduces hyperactivity in these mice^1,2^. These data suggest a serotonergic mechanism of action mediating this behavioral effect. However, microdialysis studies using fluoxetine, propose a role for cortical norepinephrine (NE) and DA^3,4^. Additionally, newer non-stimulant forms of ADHD medications (atomoxetine, guanfacine) alter NE transmission, thus providing evidence for a role for the NE transporter (NET) in this paradoxical calming effect. Interestingly, NET knockout (NET-KO) mice have reduced locomotor activity and lower striatal DA release^5^, again suggesting a crucial role for NET in this calming effect. However, NET inhibition not only elevates NE levels, but also DA levels in the PFC^4–6^. Consequently, it is still unclear whether PFC NE, DA or 5-HT are necessary for the paradoxical effects of psychostimulants.

We hypothesize that PFC DA is the common mechanism that drives this paradoxical effect. Previous microdialysis studies established an inverse relationship between DA levels in the PFC and striatal DA levels, and locomotor activity^7,8^. DA depletion by pharmacological lesioning in the PFC has been shown to increase subcortical DA levels and amphetamine-induced locomotor activity^7,9,10^. Additionally, we have previously shown that DA D2 receptor deletion in the PFC increases PCP-induced hyperactivity (Urs et al., 2016).

The present study investigated the effects of pharmacological agents that increase PFC DA levels on locomotor activity. The current study provides mechanistic insight into the regulation of PFC monoamines and their role in the paradoxical effects of psychostimulants.

## Results

**Figure 1:**
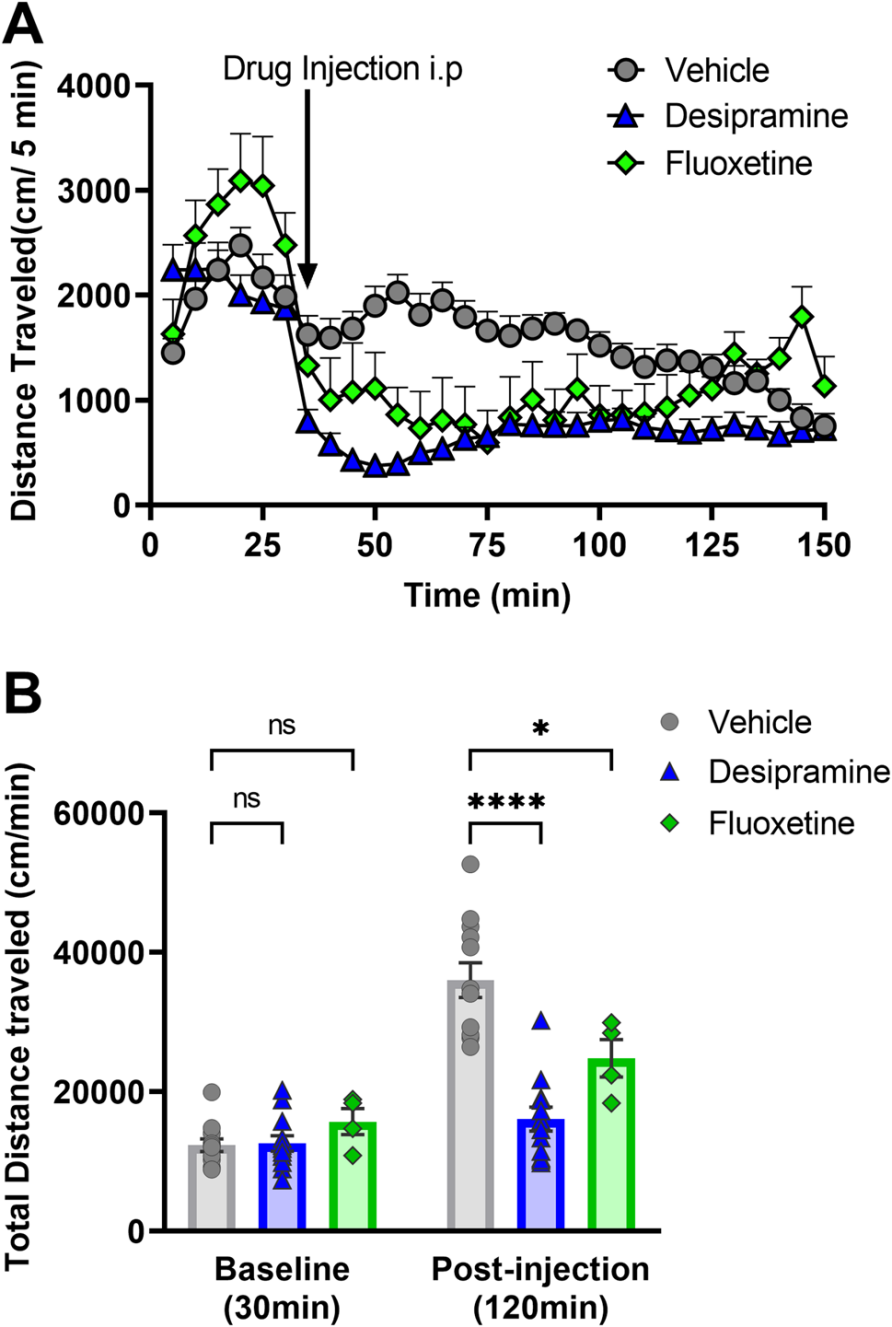
Effect of monoamine transporter drugs on DAT-KO hyperactivity. Hyperactive DAT-KO mice were systemically injected with vehicle (saline) or the transporter blockers desipramine (NET) (i.p.) or fluoxetine (SERT) (s.c). (**A)** Locomotor activity (cm/5 min) was recorded for 120 min, preceded by a 30 min baseline recording. (**B**) Total distance traveled for baseline (cm/30 min) and post-injections sessions (cm/120 min) were calculated. N = 6–12 mice per group. F (2, 26) = 23.44, *P < 0.05, ****P < 0.0001 using two-way ANOVA, compared to Vehicle-treated controls, ns- not significant.

Previous reports have shown that NET-KO mice have reduced locomotor activity^5^, whereas hyperactivity in DATKO is reduced by SERT inhibition^1^. We sought to test the effects of NET or SERT inhibition on locomotor activity in DAT-KO mice. Systemic administration of the NET inhibitor, desipramine (25 mg/kg, i.p.), or the SERT inhibitor, fluoxetine (20 mg/kg, s.c.), significantly decreased hyperactivity in DAT-KO mice, compared to vehicle (saline) injected controls (Fig. 1). Our findings are in contrast to the original study^1^, which shows that NET inhibition using nisoxetine does not affect hyperactivity in the DAT-KO mice.

**Figure 2:**
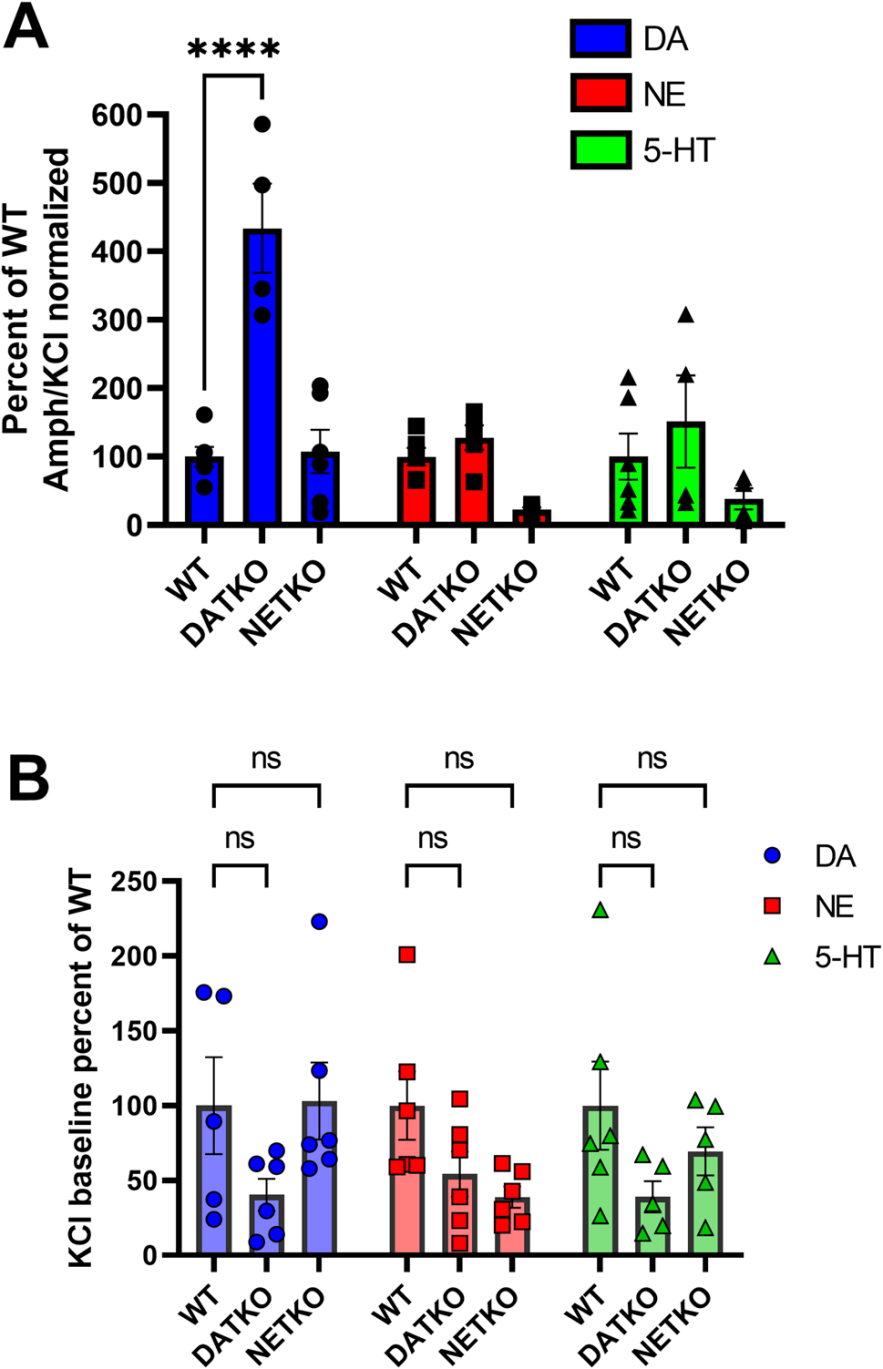
Effect of Amphetamine on monoamine efflux in PFC tissue. **(A)** PFC tissue slices from WT, DAT-KO and NET-KO mice were treated with amphetamine (AMPH, 10uM), and monoamines released into the KH incubation buffer were analyzed by HPLC. For each monoamine, Amph-induced release was normalized to KCl-induced release (shown in B) for each respective genotype and then normalized to WT levels. F (2, 42) = 7.325 for genotype comparisons. ****P < 0.0001 using two-way ANOVA, compared to WT mice. n = 2 experiments with 3 mice in each experiment. (**B)** PFC tissue slices from WT, DAT-KO and NET-KO mice were treated with 40 mM KCl, and monoamines released into the KH incubation buffer were analyzed by HPLC. For each monoamine, KCl-induced release was normalized to WT levels. *ns* Not significant using two-way ANOVA, compared to WT mice. n = 3–4 experiments with 4 mice in each experiment.

The prefrontal cortex (PFC) is thought to be the site of psychostimulant action and other therapeutics used to treat ADHD^11,12^ given that they improve motivational and cognitive functions. We asked the question if desipramine and fluoxetine alter monoamine levels in the PFC. To test this, we used a previously established ex vivo efflux assay^13,14^ that allows measurement of monoamines released from brain tissue slices submerged in oxygenated buffer. Monoamine release from the slices is induced by potassium chloride (KCl) application and measured by HPLC. KCl-mediated increases in monoamine levels can be further enhanced by application of specific monoamine transporter blockers or by drugs such as amphetamine (Amph) (see “Methods” section for details), which alter presynaptic reuptake of monoamines. We first tested Amph-induced efflux in PFC tissue slices from WT, DAT-KO, and NET-KO mice. Amph significantly elevated DA levels in DAT-KO mice, but not WT or NET-KO mice (Fig. 2A). Amph elevated NE and 5-HT to similar levels in PFC slices from WT compared to DAT-KO or NET-KO mice. Amph-induced monoamine release was normalized to baseline KCl-induced monoamine release (Fig. 2B), which did not show significant changes between genotypes. All raw values of monoamine levels in PFC tissue for Fig. 2 are shown in supplemental data Table 1.

**Figure 3:**
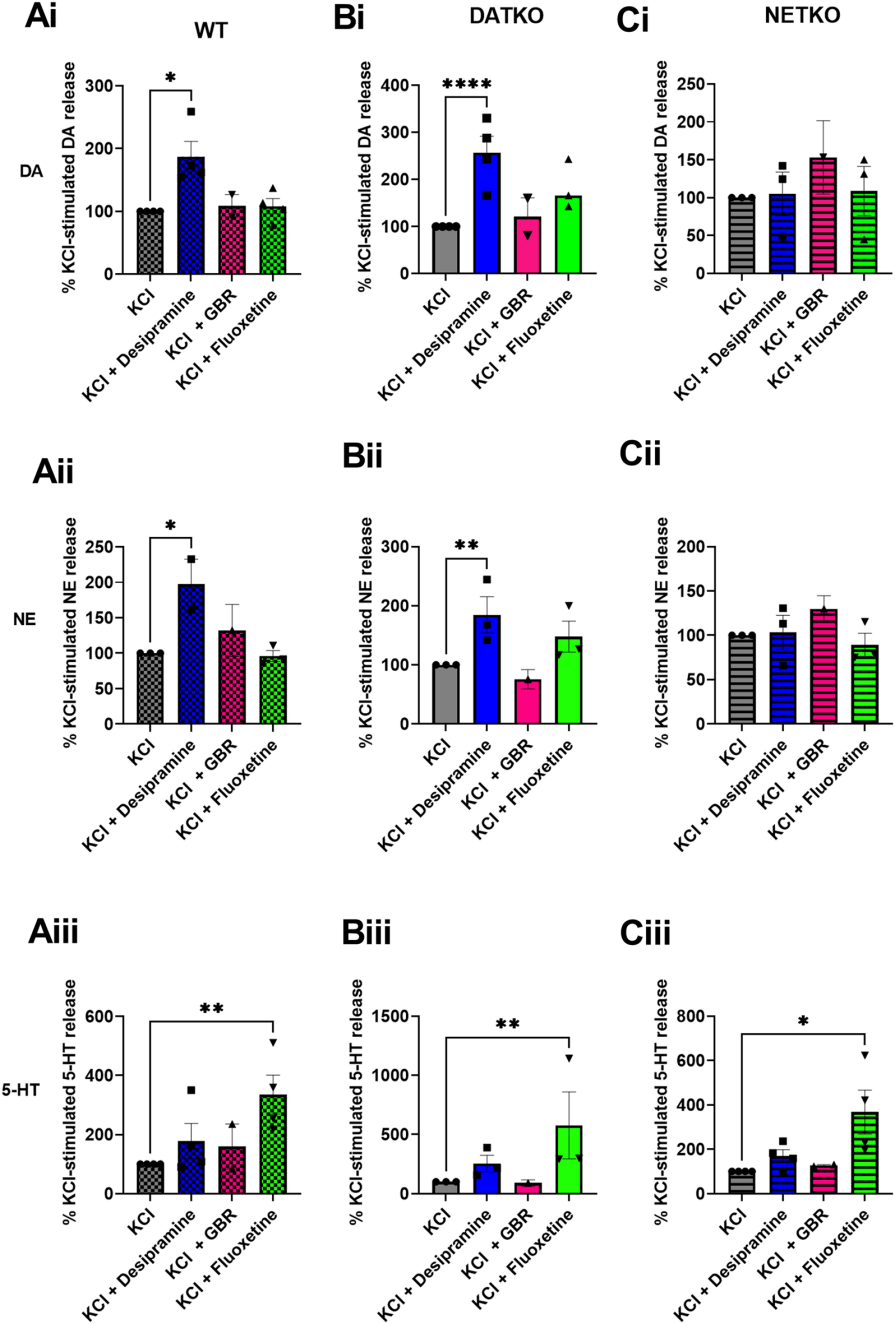
Effect of monoamine transporter blockers on monoamine efflux in PFC tissue. PFC tissue slices from WT, DAT-KO and NET-KO mice were treated with KCl alone (40 mM) or KCl + 10 µM desipramine, GBR12909 (GBR) or fluoxetine. Monoamines released into the KH incubation buffer were analyzed by HPLC. F (3, 21) = 3.712 WT NE, F (3, 34) = 3.284 WT DA, F (3, 35) = 5.957 WT 5-HT, F (3, 32) = 10.85 DAT-KO DA, F (3, 24) = 5.003 DAT-KO NE, F (3, 20) = 8.410 DAT-KO 5-HT, F (3, 24) = 0.5012 NET-KO DA, F (3, 24) = 0.4536 NET-KO NE, F (3, 35) = 3.578 NET-KO 5-HT, for drug treatment comparisons. *P < 0.05, **P < 0.01, ****P < 0.0001 using two-way ANOVA, compared to KCl-treated controls. n = 3–4 experiments with 4 mice in each experiment.

However, there was a trend towards an Amph-induced reduction in NE levels in the NET-KO mice. Amph binds to all monoamine transporters non-specifically and elevates monoamine levels by inducing reverse transport. We next tested the effects of specific transporter blockers, desipramine, GBR12909 and fluoxetine, that inhibit NET, DAT and SERT, respectively. In WT mice, NET inhibition with desipramine (10 uM) increased KCl-stimulated NE and DA but not 5-HT compared to KCl alone (Fig. 3Ai, DA; Aii NE). As expected, SERT inhibition with fluoxetine (10 uM) increased KCl-stimulated 5-HT in WT mice, but did not alter NE or DA levels (Fig. 3Aiii, 5-HT). Our findings are in contrast to previous microdialysis studies showing that fluoxetine elevates NE and DA in the PFC^3,4^. GBR12909 had no effect on any of the monoamine levels in PFC tissue slices, consistent with evidence that in the PFC DAT expression is very low compared to the striatum, and reuptake of DA is primarily mediated by NET^6,15,16^. In DAT-KO mice, NET inhibition with desipramine led to a significant increase in KCl-stimulated NE and DA, but not 5-HT levels compared to KCl treatment alone (Fig. 3Bi, DA; 3Bii, NE). The increase in DA levels in DAT-KO mice was much higher than that of WT mice (p < 0.05 vs p < 0.0001). Similar to WT mice, SERT inhibition in DAT-KO mice using fluoxetine led to a significant increase in KCl-stimulated 5-HT levels, but not NE or DA levels, compared to KCl treatment alone (Fig. 3Biii, 5-HT). Neither NET nor DAT inhibition with desipramine or GBR12909, respectively, had any effect on KCl-stimulated NE, DA, or 5-HT levels in NET-KO mice (Fig. 3Ci–iii). Conversely, SERT inhibition with fluoxetine increased KCl-stimulated 5-HT, but not NE or DA (Fig. 3Ci–iii, 5-HT).

**Figure 4:**
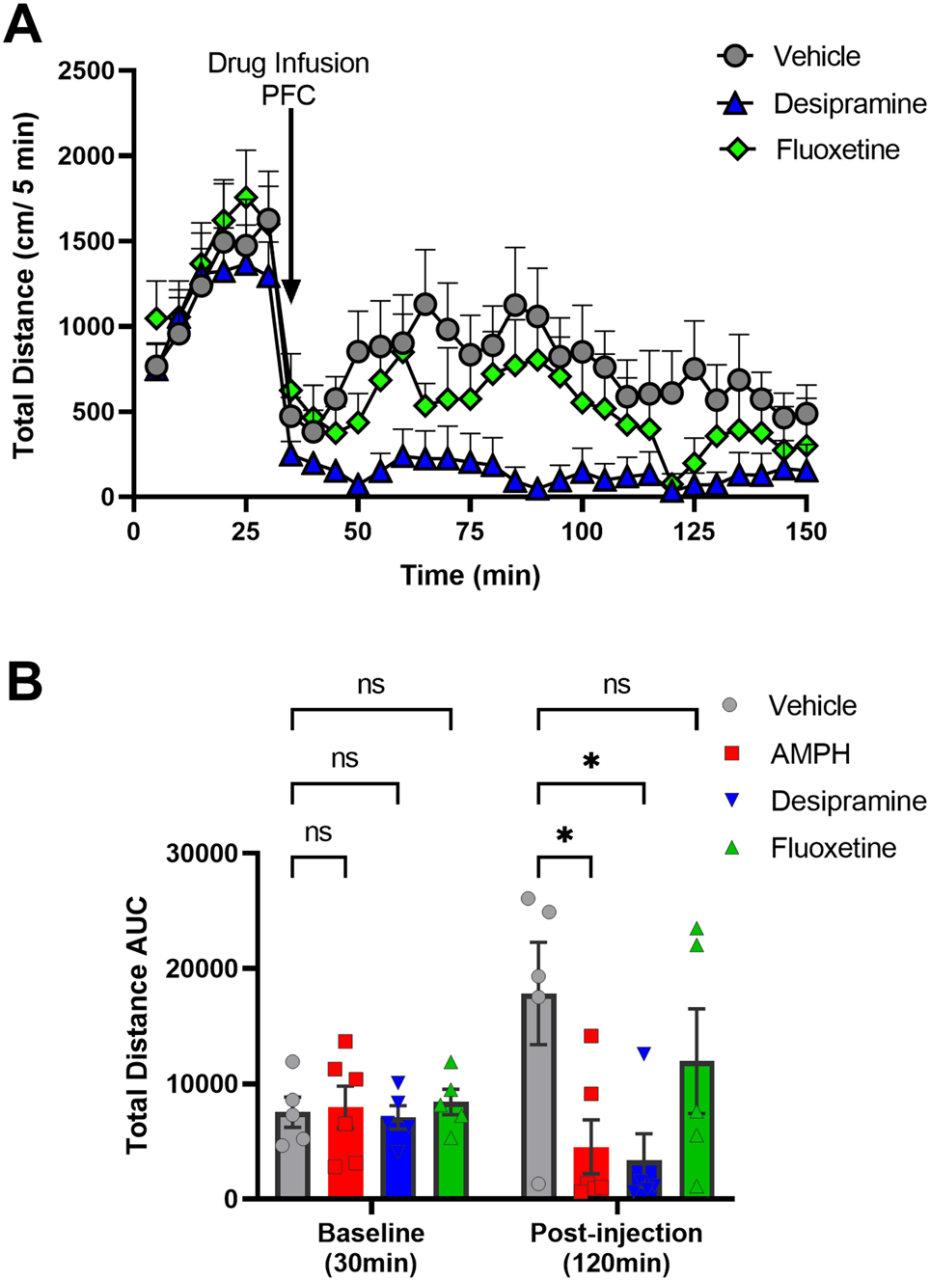
Effect of PFC infusion of monoamine transporter drugs on DAT-KO hyperactivity. **(A)** Hyperactive DATKO mice were infused (drug infusion PFC) with vehicle (aCSF) or 4ug/0.5ul of desipramine or fluoxetine (dissolved in aCSF) in the PFC, after a 30 min baseline, and locomotor activity (cm/5 min) was recorded for 120 min post-infusion. (**B)** Baseline (cm/30 min) and post-infusion (cm/120 min) total distance traveled was measured upon local infusion of desipramine, amphetamine (AMPH) or fluoxetine. n = 6–8 mice per group. Pairwise comparisons using Two-way ANOVA, F (4, 52) = 2.973 *p < 0.05, *ns* not significant.

Since our results showed that systemic NET inhibition reduces hyperactivity in DAT-KO mice and that NET inhibitors elevate both NE and DA levels in the PFC of DAT-KO mice, we next tested the effect of PFC NET inhibition on locomotor hyperactivity of DAT-KO mice. Bilateral PFC infusion of desipramine (4 µg/0.5 µl/side) significantly reduced hyperactivity in DAT-KO mice (Fig. 4A,B). Consistent with our efflux data, bilateral infusion of fluoxetine did not significantly affect hyperactivity in DAT-KO mice (Fig. 4A,B). However, bilateral infusion of Amph (100uM/0.5ul/side) significantly reduced hyperactivity in DAT-KO mice (Fig. 4B).

**Figure 5:**
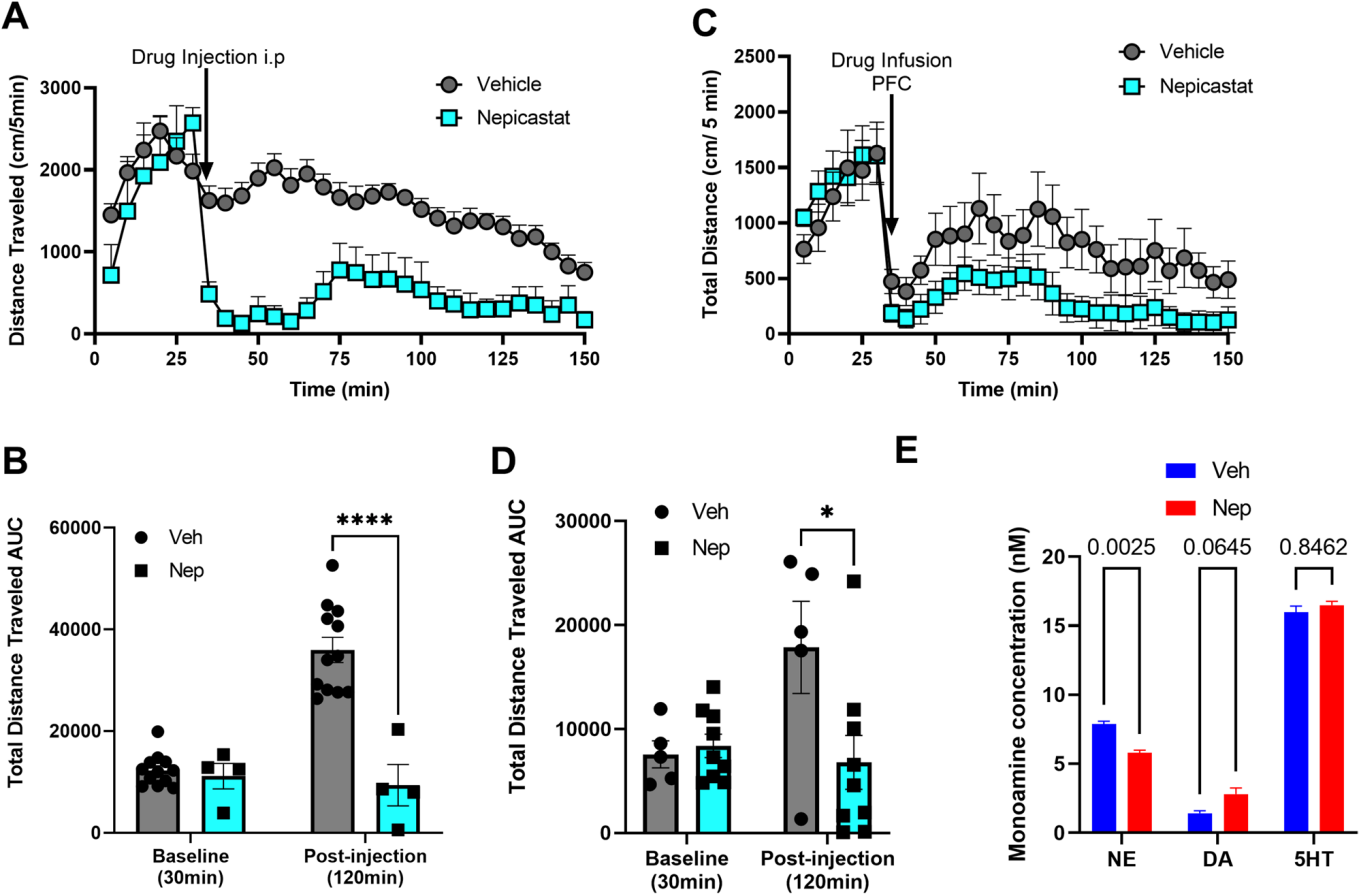
Effect of systemic or local PFC nepicastat administration on DAT-KO hyperactivity. **(A)** Locomotor activity (cm/5 min) of hyperactive DAT-KO mice was recorded for a 30 min baseline measurement followed by systemic injection with nepicastat or vehicle, and an additional 120 min of post-injection activity was recorded. (**B**) Total distance traveled (cm/150 min) was calculated. n = 6–12 mice per group. Pairwise comparisons using Two Way ANOVA, ****p < 0.0001. DAT-KO mice were infused with vehicle or 4ug/0.5ul of nepicastat in the PFC. (**C)** Locomotor activity (cm/5 min) was recorded for a 30 min baseline and an additional post-infusion recording of 120 min. (**D)** Total distance traveled (AUC) was calculated for baseline (cm/30 min) and post-infusion (cm/120 min). Pairwise comparisons using Two-way ANOVA, F (4, 52) = 2.973, *p < 0.05. (**E)** DAT-KO PFC tissue was analyzed upon nepicastat (Nep) i.p. injection for NE, DA or 5-HT levels by HPLC. P-values calculated using Two-way ANOVA, F (2, 18) = 1016, Nep compared to vehicle (veh) treated, n = 4.

Since NET inhibitors elevate NE and DA levels in the PFC of DAT-KO and reduce hyperactivity, we next sought to test the effect of NE depletion in DAT-KO mice. We tested this using the DA beta hydroxylase (DBH) inhibitor nepicastat, which depletes NE levels in the brain^17,18^. DBH metabolizes DA to NE, therefore, nepicastat not only depletes NE levels, but also enhances DA levels in the PFC^19^. Systemic administration (40 mg/kg, i.p.) (Fig. 5A,B) or bilateral PFC infusion (4 µg/0.5 µl/side) (Fig. 5C,D) of nepicastat reduced hyperactivity in DAT-KO mice. HPLC analysis of PFC tissue from DAT-KO mice systemically injected with nepicastat showed reduced tissue NE levels and elevated tissue DA levels without altering 5-HT levels (Fig. 5E).

## Discussion

In this study, we apply an integrated approach, using ex vivo efflux techniques, monoamine transporter genetic knockouts, and behavior, to understand the molecular and anatomical mechanisms that regulate the phenomenon of paradoxical calming effects of psychostimulants in ADHD therapy. We showed that the NET inhibitor desipramine, but not the SERT inhibitor fluoxetine, elevates NE and DA levels from PFC tissue slices. Importantly, local PFC infusion of the NET blocker desipramine or DBH inhibitor nepicastat inhibits hyperactivity in DAT-KO mice. These data suggest that elevated PFC DA is a common mechanism that drives the paradoxical calming effect of psychostimulants.

We observe that systemic administration of desipramine and fluoxetine reduces hyperactivity in DAT-KO mice, which are consistent with previous findings. However, our findings are in contrast with a previous study that failed to show a reduction in hyperactivity in DAT-KO mice following treatment with the NET inhibitor nisoxetine^1^. The dose of nisoxetine used in the previous study was 10 mg/kg, whereas other studies found that higher doses of nisoxetine did produce behavioral effects in DAT-KO mice^20^. The present study, as well as previous studies, thus suggest a role for NET in the paradoxical calming effects in DAT-KO mice. Our findings are consistent with a role for NET in regulating locomotor activity^5^. Thus, both NET and SERT may play a role in the paradoxical calming phenomenon of psychostimulants. However, the exact anatomical and molecular mechanisms of this phenomenon are not clear. Studies have suggested that the site of action of these psychostimulants is in the PFC, using very low doses of psychostimulants. Low dose administration of psychostimulants is thought to preferentially affect monoamine physiology through PFC, and not striatal, monoamine transporters^11^. Consistent with these studies, we observed that NE and DA levels are elevated in DAT-KO mice in the presence of the NET inhibitor desipramine. This effect is mediated by NET, since NET-KO mice did not show elevated NE and DA levels. It is possible that these effects could be due to compensatory changes due to lifelong deletion of DAT. We found no alterations in PFC tissue monoamine levels in DAT-KO mice, but significantly lower PFC NE tissue levels in NET-KO mice (Fig. S1), as shown by HPLC. These results suggest that the increase in stimulated monoamine release in DAT-KO mice are not due to homeostatic changes in the PFC. Our notion is consistent with previous studies showing that monoamine transporter expression is unaffected in DAT-KO mice, although alterations in striatal D2 receptors are observed^21^. Unlike desipramine, our efflux studies show that fluoxetine elevates only 5-HT levels and not NE or DA. This is in contrast to previous microdialysis studies showing that fluoxetine increases PFC NE and DA^3,4^. This discrepancy can be explained by the fact that we used brain tissue slices, in which the long-range connectivity of PFC afferents from midbrain or hindbrain is disrupted, whereas in microdialysis studies, this connectivity is maintained. Additionally, in our PFC infusion experiments, desipramine, but not fluoxetine, reduced DAT-KO hyperactivity. This suggests that the site of action for fluoxetine is presumably outside of the PFC, since systemic administration of fluoxetine does reduce DAT-KO hyperactivity. Recent anatomical mapping studies have suggested that serotonergic raphe nuclei send dense projections to VTA DA neurons and locus coeruleus (LC) NE neurons that project to the medial PFC^22–24^. It is likely that fluoxetine’s action on raphe terminals at mesocortical DA or LC neurons activates these neurons and their cortical projections to elevate PFC DA and NE levels, respectively. Another study suggesting a possible serotonergic mechanism administered the 5-HT precursor 5-HTP, and also showed a reduction in DAT-KO hyperactivity^1^. However, it is possible that excess systemic 5-HTP levels overload the decarboxylase enzyme that is also present in other monoaminergic neurons, such as DA neurons. Such an overload could lead to depletion of striatal dopamine, similar to administration of the tyrosine hydroxylase inhibitor alpha-methyl-p-tyrosine, which has been shown to inhibit DAT-KO hyperactivity^25^. Overall, two distinct possible mechanisms, i.e. desipramine in the PFC and fluoxetine in the mid/hind-brain, may regulate DA and NE levels in the PFC. However, PFC infusion of the DBH inhibitor nepicastat, which depletes NE while elevating DA, also inhibits hyperactivity in DAT-KO mice. Together, these data suggest PFC DA, and not NE or 5-HT, serves as a convergent signal for distinct mechanisms of NET or SERT regulation of hyperactivity in DAT-KO mice. This hypothesis is consistent with previous data suggesting an inverse role of cortical DA and striatal DA in regulating locomotor activity^7,8^.

Thus, our data provide a mechanistic insight into possible mechanisms of the paradoxical calming effects of psychostimulants and highlight a predominant role for cortical DA in this phenomenon. Our previous studies have identified a possible cortico-striatal-midbrain circuit involved in cortical DA control of striatal DA function and behaviors^26^. Future studies elucidating the circuit mechanisms regulated by this cortical DA-dependent phenomenon will provide a possible platform for studying the regulation of striatal DA dysfunction, which is a pathological feature of multiple neurological and psychiatric disorders.

## Methods

### Animals and drugs

All mouse studies were conducted in accordance with the NIH guidelines for animal care and use and with an approved animal protocol from the University of Florida Animal Care and Use Committee. Studies were conducted in compliance with ARRIVE guidelines. The DAT-KO^2^ and NET-KO^5^ mice were obtained from Dr. Marc Caron (Duke University). The knockout mice and littermate controls were backcrossed on to a C57BL6/J background for at least 10 generations and are maintained on this background. 2–5 month old mice of both sexes were used for all studies. Amphetamine (Amph, Sigma), desipramine (Sigma), fluoxetine (Sigma), and GBR12909 (R&D systems) were dissolved in saline (behavior) or efflux buffer (efflux assays). Nepicastat (Medkoo, Morrisville, NC) was dissolved in 1% DMSO and 10% hydroxypropyl-cyclodextrin (Sigma) and brought up to volume with saline. Appropriate solvent solutions were used as vehicle controls. For systemic injections, all  drugs were injected at a volume of 10 ml/kg animal weight.

### Locomotor activity

Locomotor activity was measured in an Accuscan activity monitor (Accuscan Instruments, Columbus, OH) as described previously^27^. Briefly, locomotor activity was measured at 5 min intervals, and the total distance traveled in 5 min increments for a total of 150 min or as mentioned in figures were analyzed. Mice were acclimatized to the activity monitoring chambers for 30 min prior to any drug treatments. Drugs were administered after the acclimatization period and locomotor activity was recorded for an additional 120 min.

### Ex vivo monoamine release (efflux) assay

Wild-type (WT), DAT-KO and NET-KO mice were sacrificed and brains were quickly removed and immersed in ice-cold Krebs–Henseleit (KH) buffer in the presence of 400 µM ascorbic acid. The composition of KH buffer in mM was: NaCl 116; KCl 3; MgSO_4_ 1; KH_2_PO_4_ 1.2; NaHCO3 25; D-glucose 11; pH 7.4 saturated with O_2_/CO_2_ (95/5% v:v). The brains were sliced into coronal sections (250 µm thick) using a vibratome (Leica). Coronal sections of the PFC were collected and kept in ice-cold, oxygenated KH buffer until used for testing. Sections from 4 animals/experiment were equilibrated at 37 °C in KH buffer in the presence of 1.8 mM CaCl_2_, 10 µM pargyline, a MAO inhibitor, and 400 µM ascorbic acid (efflux buffer) for 30 min. After 30 min, the slices were transferred to chambers containing efflux buffer + drug treatment (40 mM KCl + /− 10 µM desipramine, 10 µM GBR 12909, 10 µM Fluoxetine) for 20 min at 37 °C. Following incubation with efflux buffer alone or efflux buffer + drug treatment, 0.1 M perchloric acid was added to the supernatants collected from the samples. The supernatants were centrifuged for 5 min at 5000 *g*. For amphetamine-induced monoamine release, data were normalized to KCl-induced release for each genotype, respectively, to account for homeostatic changes in release due global deletion of transporters.

Monoamine levels were measured using an HTEC (Amuza Inc, San Diego, CA) HPLC system with an electrochemical detector. Monoamine release was calculated as the amount of catecholamines in the eluate normalized to total protein levels in the tissue samples and normalized to the KCl-induced control samples. Data from at least 3 independent experiments (each experiment with tissue from n = 4 mice) were pooled together.

### PFC infusions

DAT-KO mice underwent surgery to implant bilateral cannula (Plastics One, 33-gauge, 1 mm) into the PFC using stereotaxic coordinates from a mouse brain atlas (AP: + 2.4 mm, ML: 0.5 mm, DV: − 1.0 mm from bregma). Following at least 1–2 weeks of recovery, the mice were placed in an open-field testing chamber and baseline locomotor activity was assessed for 30 min. Locomotor activity was paused while the mice received a bilateral infusion (0.5 µl/side) of either desipramine, fluoxetine, nepicastat (all 4 µg/0.5 µl), amphetamine (100 µM/0.5 µl) or vehicle (aCSF or aCSF with 10% cyclodextrin, 1% DMSO for nepicastat) over a 2 min period. Locomotor activity recording was resumed for an additional 120 min. Mice were injected with either vehicle or drug and after a 72-h drug washout, were counterbalanced and the injection groups were reversed.

### Monoamine tissue level-HPLC analysis

WT, DAT-KO and NET-KO mice were sacrificed and brains were placed in a mouse brain matrix. The mouse brain matrix was used to take 1 mm coronal sections and punches of the PFC, and dorsal striatum (method described by Salvatore et al., 2012). The punches of each of the brain regions of interest were place on dry ice and stored at − 80 °C until processed. For processing, tissue samples were placed in ice-cold 0.1 M HClO4 solution and sonicated at 30% of full power. The volume of 0.1 M HClO4 solution was 20 times the weight of PFC and 40 times the weight of dorsal striatum tissue samples. The samples were subsequently centrifuged at 16,000 *g* for 15 min at 4 °C and the supernatants were placed in a separate tube from the protein precipitates. The supernatants were filtered and the filtrates were placed in HPLC tubes. Monoamine levels were determined by HPLC analysis with electrochemical detection (HTEC, Amuza Inc).

### Statistical analyses

All statistical analyses were performed using Graphpad prism 9.0. All group sizes for behavioral analyses are based on our previously published studies, as well as on power analyses suggesting that they will yield power to detect significant (alpha = 0.05) effects of 0.8 and above. Data were analyzed by unpaired t-test, One-way ANOVA, or Two-way ANOVA test for comparison between genotypes and/or injection pairs, as mentioned in figure legends. Individual genotypes, or injection pairs were compared using a post hoc Tukey’s test. Data are presented as mean ± SEM. p < 0.05 is considered as significant.

## Supplementary Information

Supplementary Information.

## Data Availability

The datasets generated during and/or analyzed during the current study are stored on a University of Florida One Drive account and available from the corresponding author on reasonable request.
