# Supplementary material for: A role for cortical dopamine in the paradoxical calming effects of psychostimulants
Source: Sci Rep. 2022 Feb 24;12:3129. doi: 10.1038/s41598-022-07029-2 (PMC8873208; doi:10.1038/s41598-022-07029-2)
Supplement: Supplementary file 1 — Supplementary Information. [file 41598_2022_7029_MOESM1_ESM.docx]

**
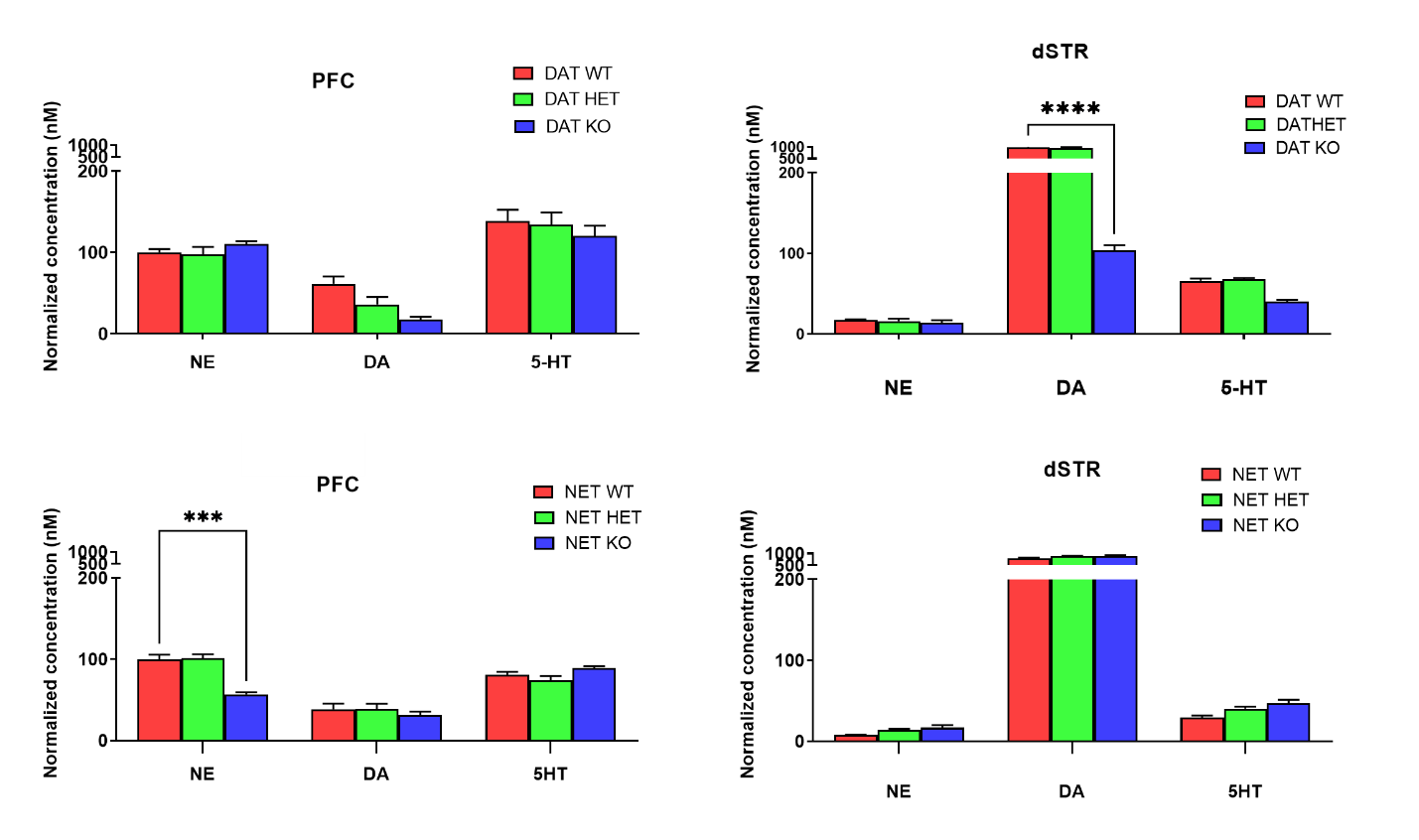
**

**Figure S1. Monoamine tissue levels in DAT-KO and NET-KO mice.** DAT-KO or NET-KO PFC and dorsal striatum (dSTR) tissue were dissected and analyzed for norepinephrine (NE) or dopamine (DA) or serotonin (5-HT) levels by HPLC. F (2, 24) = 817.1 DAT mice, F (2, 24) = 5.822 NET mice for genotype comparisons. ***P<0.001, ****p<0.0001 using Two-way ANOVA, compared to WT tissue levels n=4. Monoamine levels were normalized to PFC WT tissue levels of NE.

| **DA** | | | | | |
| --- | --- | --- | --- | --- | --- |
| **KCl** | | | **Amph** | | |
| **WT** | **DAT KO** | **NET KO** | **WT** | **DAT KO** | **NET KO** |
| 0.0021 | 0.0017 | 0.0041 | 0.0018 | N.D | 0.0065 |
| 0.0013 | 0.0008 | 0.0036 | 0.0028 | 0.0041 | 0.0069 |
| N.D | 0.0034 | 0.0043 | 0.0028 | 0.0046 | 0.0011 |
| 0.0098 | 0.0005 | 0.0069 | 0.0035 | N.D | 0.0006 |
| 0.0096 | 0.0039 | 0.0032 | 0.0035 | 0.0078 | 0.0036 |
| 0.0050 | 0.0033 | 0.0124 | 0.0053 | 0.0066 | 0.0030 |

| **NE** | | | | | |
| --- | --- | --- | --- | --- | --- |
| **KCl** | | | **Amph** | | |
| **WT** | **DAT KO** | **NET KO** | **WT** | **DAT KO** | **NET KO** |
| 0.0062 | 0.0040 | 0.0043 | 0.0026 | 0.0020 | 0.0002 |
| 0.0060 | 0.0024 | 0.0031 | 0.0023 | 0.0008 | 0.0003 |
| 0.0204 | 0.0071 | 0.0057 | 0.0028 | 0.0022 | 0.0002 |
| 0.0098 | 0.0008 | 0.0062 | 0.0035 | N.D | 0.0001 |
| 0.0061 | 0.0106 | 0.0020 | 0.0016 | 0.0015 | N.D |
| 0.0124 | 0.0082 | 0.0023 | 0.0016 | 0.0018 | N.D |

| **5-HT** | | | | | |
| --- | --- | --- | --- | --- | --- |
| **KCl** | | | **Amph** | | |
| **WT** | **DAT KO** | **NET KO** | **WT** | **DAT KO** | **NET KO** |
| 0.0006 | 0.0005 | 0.0004 | 0.0090 | 0.0041 | 0.0020 |
| 0.0004 | 0.0004 | 0.0007 | 0.0078 | 0.0050 | 0.0018 |
| 0.0017 | 0.0001 | 0.0006 | 0.0022 | 0.0036 | 0.0002 |
| 0.0006 | 0.0003 | 0.0008 | 0.0037 | N.D | N.D |
| 0.0002 | 0.0001 | 0.0001 | 0.0014 | 0.0005 | 0.0005 |
| 0.0010 | N.D | N.D | 0.0009 | 0.0007 | 0.0003 |

**Table 1. Raw values (nmol/mg) of monoamines levels in PFC tissue of WT, DAT or NET-KO mice normalized to protein levels. Values shown are from 3 different experiments with tissue pooled from 3-4 mice for each experiment. N.D not detected**
